# Micronodular basal cell carcinoma of the scrotum: a case report and review of the literature

**DOI:** 10.1186/s13256-021-03124-6

**Published:** 2021-10-17

**Authors:** Mohammad Younes, Lamia Kouba, Hanaa Almsokar, Ayham Badran

**Affiliations:** University Hospital of Dermatology and Venereology, Damascus University, Damascus, Syria

**Keywords:** Basal cell carcinoma, Micronodular, Scrotum, Case report

## Abstract

**Introduction:**

Basal cell carcinoma is the most common nonmelanotic skin cancer. It has variable clinical and histological subtypes that vary in their aggressiveness and liability to recurrence and metastasis. Chronic ultraviolet radiation exposure is considered to be the main risk factor for developing basal cell carcinoma; therefore, it typically arises on sun-exposed skin, mainly the head and neck.

**Case presentation:**

We present the case of a 55-year-old Caucasian male who presented with a lesion on the scrotum for 2 years. The lesion was clinically presumed benign and initially treated with curettage. Microscopic examination revealed an incompletely resected micronodular basal cell carcinoma with sebaceous differentiation. Therefore, a second excisional biopsy was performed to completely excise the incidentally discovered malignant tumor.

**Conclusion:**

We report the first case of micronodular basal cell carcinoma arising on the scrotum. The goal of our article is to draw clinicians’ attention to the possible involvement of unexposed skin with basal cell carcinoma, and we highlight the importance of accurate diagnosis and prompt treatment due to the aggressive nature of micronodular basal cell carcinoma.

## Introduction

Nonmelanotic skin cancer (NMC) is the most common cancer in the world. Basal cell carcinoma (BCC) and squamous cell carcinoma (SCC) represent 99% of all NMCs, with BCC being the most prevalent. However, accurate data about their prevalence are scarce, mainly because they are not reported separately in national cancer registries and many cases are not fully tracked due to the successful treatment of the tumor via surgery or ablation [1, 2].

BCC usually arises on chronically photoexposed areas in the elderly; it has been rarely reported to occur on unexposed skin such as the trunk or genitalia.

We report the case of a 55-year-old man who presented with a tumor-like lesion on the scrotum for 2 years. The lesion was excised and subsequently determined to be a micronodular BCC of the scrotum. To the best of our knowledge, this is the first reported case of micronodular BCC occurring on the scrotum.

## Case presentation

A 55-year-old Caucasian man presented to the outpatient clinic with a soft lesion on the left side of the scrotum, present for 2 years.

**Fig. 1:**
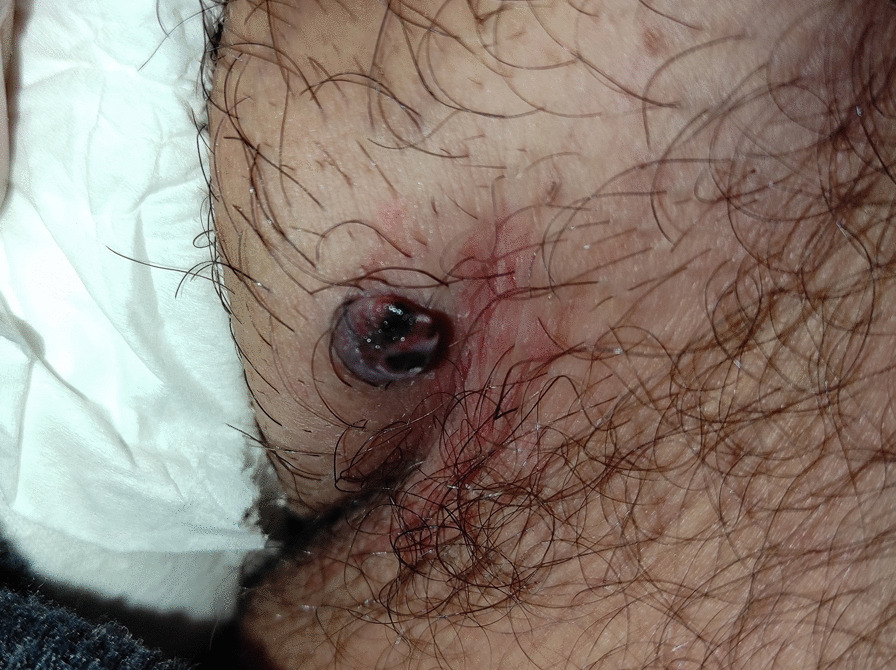
A pigmented nodule on the left scrotum measuring 7 mm in diameter

On inspection, the lesion appeared as a bluish-black nodule with rolled edges and a smooth surface. It measured 7 mm in diameter and was raised 4 mm above the surrounding skin level (Fig. 1).

According to the patient, the nodule was not painful, but due to its location in an intertriginous area that is liable to continuous friction and moisture, the lesion was prone to recurrent irritation leading to oozing, maceration, and foul odor.

The lesion started as a punctate black macule on the left side of the scrotum. The patient made several failed attempts to remove it with a razor blade.

The rest of the physical examination was unremarkable, and no lymphadenopathy was present.

Based on the patient’s history, physical examination, and the location of the lesion, the lesion was suspected to be an angiokeratoma. Consequently, it was removed by shave biopsy and sent for microscopic examination.

**Fig. 2:**
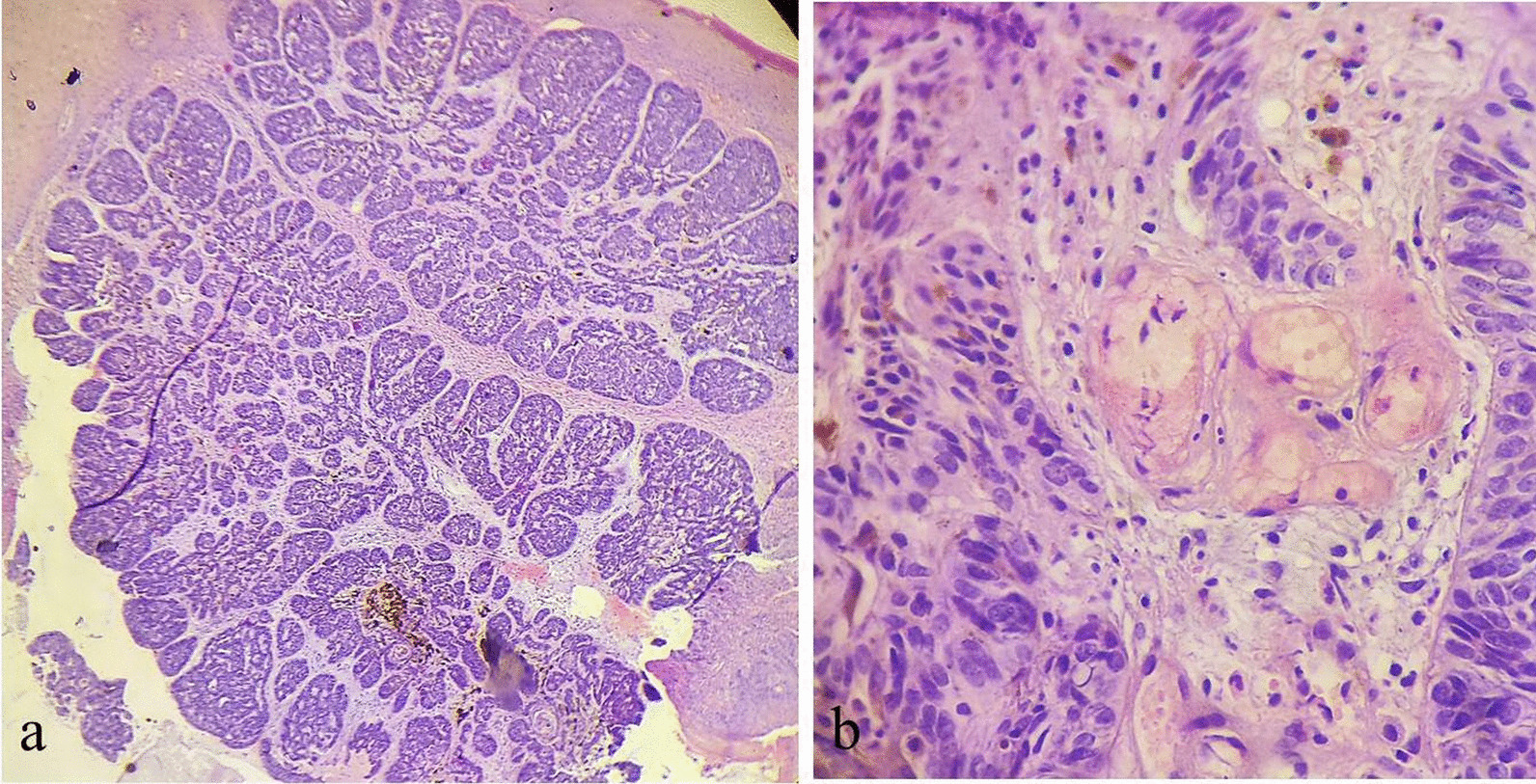
Microscopic view (hematoxylin and eosin stain) of the lesion showing **a** aggregates of small nests of basaloid cells with absent retraction artifacts. Melanin granules (brown pigment granules) can be easily seen within and outside the basaloid nests (low-power magnification). **b** High-power microscopic view depicting clusters of basaloid cells with sebaceous duct-like formations consisting of vacuolated cells with foamy cytoplasm, suggestive of sebaceous cells

Microscopic examination revealed small nests of basaloid cells extending from the epidermis and infiltrating the reticular dermis (Fig. 2a). Peripheral palisading of the nuclei was minimal, and retraction artifact was almost absent.

High-power magnification revealed multiple basaloid cells with large hyperchromatic nuclei and numerous mitotic figures. Worthy of notice is the presence of heavy pigmentation within the tumor nests and the melanophages in the surrounding stroma.

Furthermore, multiple foci of sebaceous differentiation were noted within the basaloid nests (Fig. 2b).

These microscopic findings led to the diagnosis of scrotal pigmented micronodular BCC with sebaceous differentiation.

The deep surgical margin was positive for malignant cells; therefore, the patient underwent a subsequent surgical procedure to completely excise the tumor.

On follow-up, 4 months later, no signs of recurrence were noted.

## Discussion

Basal cell carcinoma is the most frequently occurring cancer in humans. It arises from the basal layer of the epidermis and grows slowly over multiple years.

Key risk factors for developing BCCs have been recognized, including ultraviolet radiation, fair complexion, chronic arsenic exposure, ionizing radiation, personal or family history for BCC, and genetic predisposition [3, 4].

In our case, the patient had no personal or family history of BCC and no prior exposure to ionizing radiation or other carcinogens. The location of the carcinoma on the scrotum in our case renders ultraviolet exposure an unlikely culprit.

Basal cell carcinoma has multiple histological subtypes, and they can be classed according to their risk of recurrence into low-risk and high-risk subtypes. The nodular, superficial, fibroepithelial, pigmented, and infundibulocystic BCC are classified as low-risk subtypes, while the infiltrative, micronodular, morpheaform, and basosquamous BBC as well as BCC with sarcomatoid differentiation are considered as the higher-risk subtypes [5]. However, histological patterns may overlap.

Nodular BCC is the most common variant, characterized clinically by rolled edges, surface telangiectasia, and a central ulcer, giving rise to what is known as the *rodent ulcer*.

The micronodular variant is an aggressive type of BCC that is liable to recurrence and difficult to eradicate. It occurs most frequently in the head and neck area [6]. Clinically, micronodular BCC typically presents as a poorly defined infiltrated flat lesion that rarely ulcerates.

Approximately 80–85% of BCC occur on the head and neck, while 15% develop on the trunk [7]. According to a classic review conducted by Rabbari and Mehregan, less than 0.5% of BCCs were located in the genital area [8].

Less than a hundred cases of BCC arising on the perianal and the genital area have been reported in the literature [9].

Solimani and colleagues reported three cases of nodular BCC on the scrotum occurring during a span of 10 years in their institution [10], whereas Chen *et al*. conducted a recent population-based analysis of genital BCCs and identified 255 male cases, of which 190 had scrotal BCC (74.5%). An interesting finding was that penile BCC had poorer prognosis than scrotal [11].

We highlight herein 14 case reports of scrotal BCC, reported over the past 20 years; the patients’ details, tumor morphology, and microscopic classification are summarized in Table 1.

**Table 1:** Case reports published in the past 20 years of BCC arising on the scrotum

Refs.	Year	Authors	Country	Patient age	Morphology	Pigmentation	Size (cm)	Microscopic type	Metastasis	Months to presentation	Carcinogen exposure
[12]	2000	Takahashi *et al*.	Japan	49	Hyperkeratotic erythematous plaque	No	1	–	No	12	No
[13]	2000	Vandeweyer *et al*.	Belgium	66, 71, 58, 74	Ulcer with pearly border, erythematous plaque	No	0.5, 1.5, 0.9, 1.5	Solid BCC	No	9	History of radiation exposure
[14]	2002	Chave *et al*.	UK	69	Nodule with central ulcer	Side pigmentation	1.5	–	No	3, 6	No
[15]	2002	Ribuffo *et al*.	Italy	75	Ulcer	No	–	–	Perineal skin	60	No
[16]	2004	Izikson *et al*.	USA	77	Ulcerated nodule	Variegated	4	Nodular BCC	No, recurrence +	–	Coal tar, asbestos, machine oil, sulfur, hydraulic fluid, (smoker)
[17]	2005	Kinoshita *et al*.	Japan	80	Ulcerated nodule	No	2.5	–	LN, recurrence	96	No
[18]	2008	Ouchi *et al*.	Japan	54	Pedunculated nodule	Yes	1.7	Polypoid BCC	No	6	No
[19]	2008	Rao *et al*.	India	75	Ulcerated nodule	Yes	4	–	No	24	No
[20]	2011	Jianwei *et al*.	China	74	Ulcer with pearly border	No	2	Nodular BCC	No	612	Benzene
[21]	2014	Li *et al*.	China	61	Eroded plaque, rolled border	No	4	Nodular BCC	No	18	No
[22]	2016	Delto *et al*.	USA	69	Fungating verruciform mass, flat lesion	No	10	–	No	–	NF, (smoker)
[23]	2016	Hernandez *et al*.	Spain	50	Eroded exophytic tumor	No	1	Solid BCC	No	12	Asbestos
[24]	2018	Padoveze *et al*.	Brazil	87	Perlaceous tumor with telangiectasias	No	2.5	Nodular BCC	No	6	No
[25]	2020	Han *et al*.	China	74	Nodule	No	2	Superficial BCC	No	144	No
	2021	Current case	Syria	55	Nodule	Yes	0.7	Micronodular BCC	No	24	No

*LN* lymph nodes, *NF* neurofibromatosis

The average age of patients was 67.6 years (49 87 years), and the most commonly reported clinical morphology was ulcerated nodule with pearly borders. The average age of the lesion at presentation was 6.5 years (3 months to 51 years).

Unlike our case, the reported lesions were infrequently pigmented at presentation.

To our knowledge, there are no reported cases of micronodular BCCs arising from the scrotal dermis. Our article is thus the first reported case of such a rare location and histological type.

## Conclusion

The presence of BCC in an unusual anatomical location represents a diagnostic challenge for clinicians. Our report adds to the growing body of literature on the unusual sites of basal cell carcinoma. Although the majority of BCCs occur in sun-exposed areas, a diagnosis of BCC should never be excluded merely due to the absence of sun exposure. Clinicians need to be aware of the variable morphologic features of BCC and its possible occurrence in unusual sites, such as the genital area. Prompt diagnosis and proper treatment of BCC is crucial to spare the patient long-term consequences and preserve appropriate quality of life.

## Data Availability

Not applicable.

## Abbreviations

BCC: Basal cell carcinoma
NMC: Nonmelanotic skin cancer
SCC: Squamous cell carcinoma
